# The Prevention of Syphilis

**Published:** 1882-02

**Authors:** 


*The Prevention of Syphilis.*—By J. William White, M. D. Lecturer on Veneral Diseases in the University of Pennsylvania, etc , etc.

This is a published address which was delivered before the Philadelphia County Medical Society and prepared by request, being reprinted from the *Phil. Med. Times*, of January, 1882. The subject discussed is of wide interest, and the article shows careful and comprehensive investigation; and the results of stated examinations of prostitutes as instituted by the authorities of some of our cities, are given as a proof of the beneficial effects of such a method of surveillance.

